# Comparison of Endoscopic Variceal Ligation with Endoscopic Sclerotherapy for Secondary Prophylaxis of Variceal Hemorrhage: A Randomized Trial

**DOI:** 10.7759/cureus.2977

**Published:** 2018-07-13

**Authors:** H Sakthivel, Ashok Kumar Sahoo, Sakthivel Chinnakkulam Kandhasamy, Anandhi Amaranathan, Mangala Goneppanavar, Vishnu Prasad Nelamangala Ramakrishnaiah

**Affiliations:** 1 Senior Resident, Jawaharlal Institute of Postgraduate Medical Education and Research (JIPMER), Puducherry, IND; 2 General Surgery, Jawaharlal Institute of Postgraduate Medical Education and Research (JIPMER), Puducherry, IND; 3 Surgery, Jawaharlal Institute of Postgraduate Medical Education and Research (JIPMER), Puducherry, IND; 4 Pathology, Mahatma Gandhi Medical College and Research Institute, Puducherry, IND

**Keywords:** bleeding esophageal varices, endoscopic variceal ligation, eradication of varices, endoscopic sclerotherapy

## Abstract

Background

Though endoscopic variceal ligation (EVL) is commonly being used and has overcome the disadvantages of sclerotherapy (ST), still sclerotherapy is used as a therapeutic procedure for bleeding esophageal varices in the present institute. Hence, the study was done to see the advantages of EVL over ST.

Methods

Patients with portal hypertension and bleeding esophageal varices underwent banding if found to have grade 3 or 4 varices. They were randomized to EVL group, where they were reviewed after two weeks for any residual varices for which repeat banding was done and endoscopic sclerotherapy (EST) group, where ST was done until the varices were obliterated or reduced to grade 1. The efficacy, complications, recurrent bleeding rate and recurrence of varices were compared.

Results

A total of 60 patients were included, 30 in each group. In EVL group, four sessions were needed to eradicate the varices in 73% of patients while it was five sessions in EST group (46% patients) (p-value = 0.0001). The mean number of sessions needed in EVL and EST group was 3.73 and 5.36, respectively. The average time taken for eradication of varices was 78.6 and 134.6 days in EVL and EST group, respectively (p-value = 0.004). Complications were higher in EST group (p-value < 0.05).

Conclusion

EVL alone was effective than ST in terms of the number of sessions needed for eradication of varices and total duration required to completely obliterate them. The complications were less in EVL group with no significant difference in recurrent bleeding rate and recurrence of varices between the groups.

## Introduction

The life-threatening complication of portal hypertension is gastroesophageal variceal haemorrhage. Historically, the overall mortality is reported to be 30–50% and one-year mortality rate is as high as 70%. The frequency of variceal haemorrhage occurs at the rate of 5–15% and the strongest predictor of the bleeding is the size of varices. Larger varices have a higher risk of bleeding than smaller varices [1].

Endoscopic sclerotherapy (EST) and endoscopic variceal ligation (EVL) are the two most common endoscopic techniques used for the management of oesophageal varices. EST has largely been replaced by EVL in the management of bleeding varices [2].

EST controls bleeding in 80–90% of the patients. Faster eradication of varices is possible with EVL. The complications associated with EVL are also found to be less in comparison to other modalities. But, the limitations of EVL are:

1) Small varices cannot be obliterated after a few sessions of EVL due to technical difficulty.

2) Varices tend to recur because of the persistence of perforating veins and paraesophageal collaterals.

A meta-analysis comparing EST and banding concluded that rebleed rate, mortality rate and the rate of deaths due to bleeding are decreased in the ligation group [3]. EST alone for secondary prophylaxis of variceal haemorrhage is associated with more complications [3]. Though EVL is commonly being used and has overcome the disadvantages of ST, still the ST is also used as a therapeutic procedure for follow-up of varices. After the index endoscopy with EVL, subsequent size of varices decreases in most of the patients. It has been seen that due to the reduced size of varices, the application of EVL subsequently becomes difficult while, the same may not be true for ST. Hence, the study was taken up to compare the efficacy, potency and side effects of EVL with that of ST after the reduction of the size of the varices.

## Materials and methods

The study was conducted in the department of surgery at a tertiary care referral centre over a period of 23 months between September 2012 and July 2014. The study was approved by the Ethics Committee of the Institute and has been performed in accordance with the ethical standards laid down in an appropriate version of the Declaration of Helsinki (as revised in Brazil 2013). This trial has been registered in Clinical Trial Registry – India (CTRI/2016/11/007483).

All consecutive patients presenting with acute upper gastrointestinal bleeding due to portal hypertension and grade 3 or 4 bleeding esophageal varices were included in the study. Patients with gastroesophageal varices, past history of endoscopic therapy or surgical management for varices, left-sided portal hypertension, malignancy or any other organ failure (cardiac failure, renal failure and respiratory failure) were excluded from the study.

The study was designed as prospective, open-labeled, parallel arm randomized controlled trial. The two arms were randomized in a 1:1 ratio using the block randomization method. Patients presenting to the emergency department with hematemesis were first resuscitated and then clinically assessed for the cause of portal hypertension. Blood samples were sent for investigations such as complete hemogram, renal function test, liver function test, prothrombin time and serology tests for human immunodeficiency virus (HIV), hepatitis B surface antigen (HbsAg) and anti-hepatitis C virus (anti-HCV). Ryle’s tube insertion was done for gastric decompression and also to look out for active bleed. Sengstaken–Blakemore tube was inserted in patients having active bleed. Urinary catheterisation was done in all patients. Fluid resuscitation was done using crystalloids, colloids and packed cell transfusion.

Patients with coagulation alteration with increased prothrombin time were given fresh frozen plasma till the prothrombin time was corrected to the normal range. Ultrasonography of abdomen was done to look for coarse echoes in liver, portal vein diameter, size of the spleen, presence of free fluid in abdomen and evidence of any echogenic thrombus in portal vein. Fluid resuscitation, correction of coagulation abnormality, vasoactive therapy were given as appropriate.

Endoscopic intervention was done in the endoscopic unit of the department of surgery after getting informed consent on the immediate next day of admission once the patient became hemodynamically stable. The endoscopies were performed by the expert faculties of four surgical units who first performed a diagnostic endoscopy (PENTAX company, Tokyo, Japan) to evaluate the severity and location of varices. The varices were graded based on Paquets classification into four grades. All patients who had grade 3 or 4 varices initially underwent EVL using a multiband ligator. Depending upon the grade of varices, six bands or less of COOKS company (San Francisco, CA) was applied starting from gastroesophageal junction and progressing upward 5-6 cm in a spiral manner during the first session of endoscopy. The minimum distance between two bands was 2 cm. After the initial banding session, octreotide was tapered and propranolol was started as a part of secondary prophylaxis of variceal haemorrhage following which the patients were randomised into EVL group or EST group using sealed envelope technique.

In EVL group, after the initial endoscopic banding, patients were reviewed after two weeks to look for any residual varices. If any residual varices were found, then repeat banding was done. In EST group, following the index endoscopy with banding, patients were followed up every two weeks and sclerotherapy was done using intravariceal or paravariceal injection of 2 ml of 5% ethanolamine oleate. This was continued till the varices were obliterated or reduced to grade 1. Once the varices were eradicated, patients were called in after three months for surveillance endoscopy.

The primary endpoint of the study was obliteration of the varices. Complications like superficial ulcers, deep ulcers, and esophageal stricture were assessed. The following parameters were also studied in all patients: Child-Pugh score, hemodynamic status at the time of presentation (pulse rate, blood pressure), renal and liver function tests, serology (HbsAg, anti-HCV), ultrasound abdomen features (liver size and architecture, portal vein diameter, spleen size, free fluid abdomen), aetiology of portal hypertension (cirrhosis, extrahepatic portal vein thrombosis, non-cirrhotic portal fibrosis). Variceal eradication was defined as if they were disappeared (optimal sclerosis) or if grade 1 varices were achieved. Recurrent bleed was diagnosed as bleeding after the first endotherapy before the varices were eradicated [4].

Statistical analysis and sample size calculation

As the study was done as a research of a postgraduate who had a fixed study duration of two years and the sample size to get an acceptable power of the study was found to be high, a decision was made to include all the patients encountered during the prefixed study period. According to the above statement, the total number of patients recruited for the study were 72 from which, 30 in each group were included according to the inclusion criteria with exclusion of 12 patients. Chi-square test, independent sample t-tests, and one-way ANOVA tests were used to compare variables. Findings were expressed as mean ± standard deviation; p-values less than 0.05 were accepted as statistically significant.

## Results

**Figure 1:**
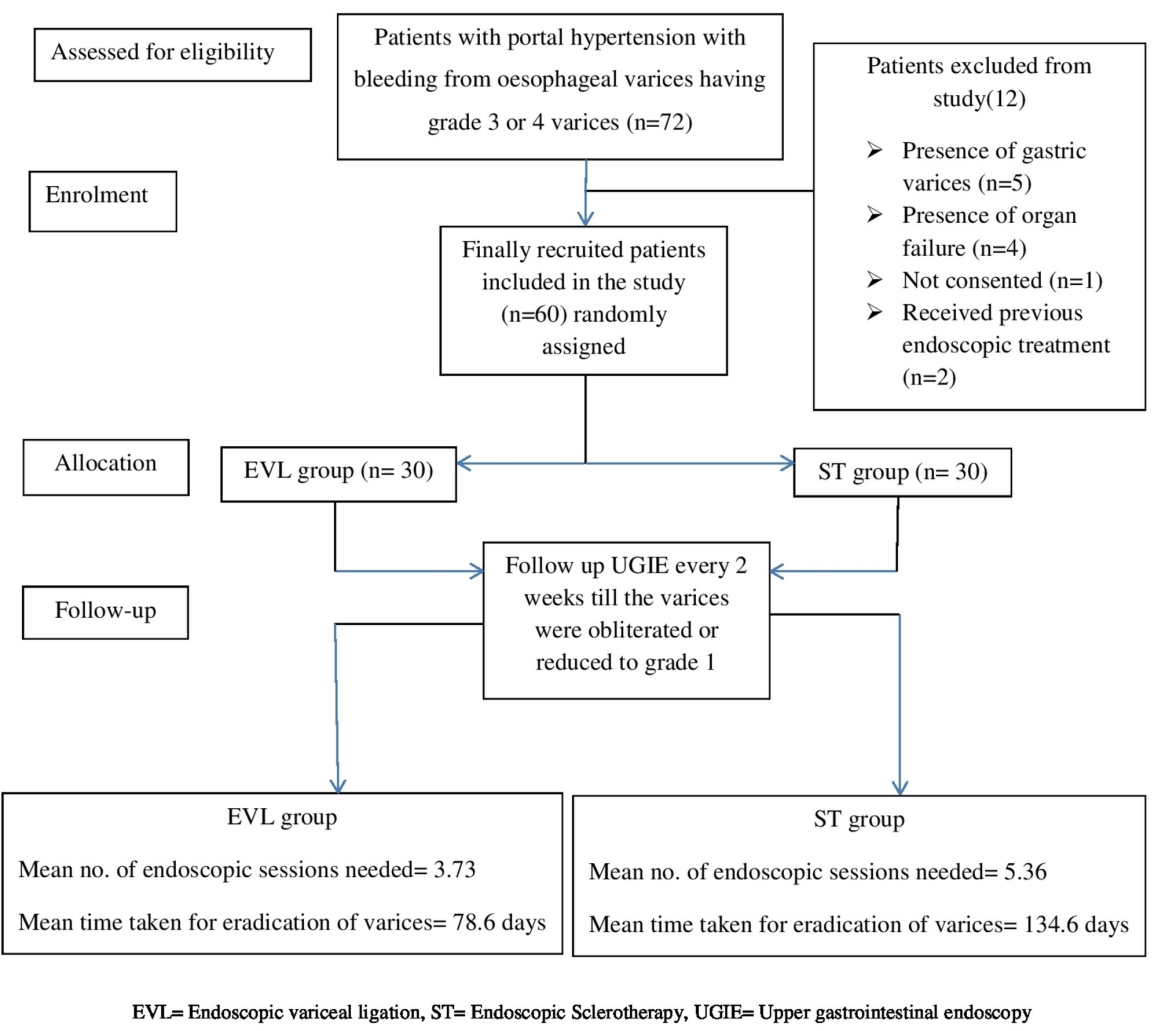
The overall scheme as per CONSORT flow chart.

In the present study, out of total 72 eligible patients, 60 patients were included in the study and were randomised into EVL group and EST group, 30 in each arm (Figure 1: CONSORT flow diagram).

The 12 patients excluded from the study were due to the presence of gastric varices (n = 5), the presence of organ failure (n = 4), not consented (n = 1), received previous endoscopic treatment (n = 2). Demographic parameters, aetiology of portal hypertension, and Child-Pugh class were comparable between both the groups (Table 1).

**Table 1:** Clinical profile of the patients in the two groups. HbsAg: Hepatitis B surface antigen.

Patient Characteristics	Endoscopic Variceal Ligation (n = 30)	Endoscopic Sclerotherapy (n = 30)
Age (years)	47.2 ± 13.6	47.7 ± 12.2
Jaundice	8 (26.7%)	5 (16.7%)
Pallor	24 (80%)	28 (93.3%)
Splenomegaly	21 (70%)	19 (63.3%)
Mean haemoglobin (gm %)	7.4	7.7
Mean platelet count (cells/ cu. mm)	1.18 lakhs	1.17 lakhs
Etiology		
NCPF (non-cirrhotic portal fibrosis)	0	3
Cirrhosis	30	27
Initial grade of varices		
Grade 3	27	28
Grade 4	3	2
HbsAg positivity	2	2
Child grade		
A	25	25
B	3	4
C	2	1

The mean age of patients in EVL group and EST group was 47.2 ± 13.6 and 47.7 ± 12.2 years, respectively. There were 19 males (63.3%) and 11 females (36.7%) in EVL group while there were 22 males (73.3%) and eight females (26.7%) in EST group. The gender distribution between the groups was also comparable and the male to female ratio in both the groups did not vary significantly (63/37% vs. 73/27%; p-value = 0.06).

In EVL group, all the patients showed features of cirrhosis on ultrasonography (USG) study while in EST group, 90% had cirrhosis with rest having features of non-cirrhotic portal fibrosis (NCPF). There was no statistically significant difference between the two groups regarding the USG finding of liver (p-value = 0.204).

The majority (83%) of patients belonged to CHILD A category followed by CHILD B category (12%). Only 5% of the study patients belonged to CHILD C category. The distribution of CHILD score categories was found to be comparable between the two groups.

It was found that among the total of 60 patients only one patient had recurrent bleed and that patient belonged to the EST group. The recurrent bleed rate was found to be statistically insignificant (p-value = 0.313). The number of sessions needed to eradicate the varices was observed in both the groups. It was found that the majority of the patients (73%) in the EVL group had their varices eradicated in four sessions and the remaining 27% required three sessions. No patient needed more than four endoscopic sessions in the EVL group. In the EST group, 46% required five sessions and 37% required six sessions for eradication of varices. Only one patient in the EST group had variceal eradication within three sessions. The mean number of endoscopic sessions needed in EVL group was 3.73 while it was 5.36 in EST group. The comparison of the number of sessions needed to eradicate the varices in the two groups was found to be statistically significant (p-value = 0.0001). The time taken for eradication of varices was observed in both the groups. The majority of patients (70%) in the EVL group had variceal eradication in less than 100 days. This was in contrast to the EST group in which the majority of patients (63%) had variceal eradication in more than 100 days. The mean number of time taken for eradication of varices was 78.6 and 134.6 in EVL and EST group, respectively. The analysis was found to be statistically significant (p-value = 0.004) (Table 2).

**Table 2:** Clinical outcome.

Clinical Outcome	Endoscopic Variceal Ligation (n = 30)	Endoscopic Sclerotherapy (n = 30)
Number of endoscopic sessions for eradication of varices (mean)	3.73	5.36
Time taken for eradication of varices (mean) (days)	78.6	134.6
p-value	<0.05	<0.05

Complications such as superficial ulcer, deep ulcer and stricture formation were observed during the follow-up endoscopy. Superficial ulceration, fever and retrosternal pain were the commonly observed complications and the analysis was found to be statistically significant (p-value < 0.05). One patient in the EVL group and two patients in the EST group developed asymptomatic mild pleural effusion which did not need any intervention (Table 3).

**Table 3:** Complications.

Complications	Endoscopic Variceal Ligation (n = 30)	Endoscopic Sclerotherapy (n = 30)	p- value
Superficial ulcers	1	9	<0.05
Fever	2	10	<0.05
Retrosternal pain	3	10	<0.05
Pleural effusion	1	2	>0.05

No patients developed strictures during the follow-up period.

## Discussion

Oesophageal variceal haemorrhage is one of the most important causes of morbidity and mortality in patients with parenchymal liver disease and portal hypertension. Once the patient is adequately resuscitated, endotherapy has become the treatment of choice for the management of bleeding oesophageal varices. After the initial bleeding episode is controlled with endotherapy, there is a substantial risk of rebleed. Patients who survive the bleeding episode should undergo repeated endoscopic treatments till the varices are obliterated to prevent recurrent bleed [5]. Variceal ligation and sclerotherapy are two common methods available for endotherapy. Though variceal ligation has been found to be the most effective method for eradication of oesophageal varices, sclerotherapy is also commonly used for the management of oesophageal varices. The present study tried to assess the efficacy, complications, and recurrent variceal bleeding between EVL with that of ST.

Age was considered as an important risk factor for variceal rebleed. In the present study, the overall mean age in the EVL group was 47.3 years while it was 47 years in EST group. More than half the patients in both groups were females; however, the gender distribution did not show any significant difference.

Lo et al. compared EVL with EST in their study. They used sclerotherapy for initial management of bleeding varices. The rate of haemorrhage control was 94% with EVL group and 80% with EST group (p-value = 0.23) [6]. In the present study, we modified it in such a way that all patients who were presenting with acute variceal haemorrhage should receive EVL for better haemorrhage control following which patients were randomised to either EVL or EST groups till the varices were eradicated. The control of bleeding was achieved in all patients.

A Turkish study conducted by Kuran et al. compared EST, EVL and combined therapy [7]. A total of 181 patients were recruited during the study period and were followed up for a period of more than six months (6-123 months). The number of sessions needed to eradicate varices was significantly less (p-value < 0.05) in EVL group (2.5 ± 1.6) when compared to the combined group (6.8 ± 3.5). The recurrent bleed rate was more in the combined group (21%) when compared to EVL group (7%) but it was not statistically significant. The study also showed that the overall rate of complications was significantly lower in the EVL group when compared to the combined group. The complications encountered in the combined group during the study were development of portal hypertensive gastropathy, fundal varices, bleeding, ulcer and stricture formation. In the present study, we found that the average number of endoscopic sessions needed to eradicate varices was 5.4 in the EST group whereas in the EVL group, it was 3.7 and the difference was found to be statistically significant (p-value = 0.0001). Only one patient in the present study had recurrent bleed and he belonged to the EST group but it was statistically insignificant. The most common complications encountered in the present study were superficial ulceration, fever and retrosternal pain which was seen more commonly in the EST group and was found to be statistically significant (p-value < 0.05).

In 1999, a study from Saudi Arabia was done to compare combined therapy with EVL alone [8]. The study was conducted with a sample size of 60 patients with 31 patients receiving EVL alone and 29 patients receiving combined therapy. There was no significant difference between the two treatment groups with respect to the number of endoscopic sessions needed to eradicate varices, recurrence of varices, complications and death, and it was recommended EVL alone for the eradication of varices. In the present study, the majority of patients were alcoholic cirrhotics and the number of sessions and days needed to eradicate varices was significantly less in the EVL group when compared to the EST group. Also, the complications observed in the EST group were significantly higher when compared to EVL alone (p-value < 0.05). The study conducted by Svoboda et al. compared EVL and EST in liver cirrhosis with esophageal varices but with no previous history of upper gastrointestinal haemorrhage [9]. The rate of eradication of varices was similar between both the groups (81% in the EVL group versus 85% in the EST group). The mean number of sessions needed for eradication of varices was lower in the EVL group than in the EST group (4.8 ± 1.8 versus 6.2 ± 2.0; p-value = 0.0003). However, the EVL group had more number of recurrence of esophageal varices than EST group (31% versus 11%; p-value = 0.01). A significant decrease in variceal bleeding was observed both in sclerotherapy cases (20%) and controls (54%; p-value = 0.0005) and in ligation cases and controls (29%; p-value = 0.01). He concluded that sclerotherapy and variceal ligation are equally effective in the prevention of first variceal bleed. In the present study, we compared EST and EVL after the initial episode of banding in all patients. We found that the number of sessions required to eradicate varices was less in EVL group, but the recurrence of varices was not found to be statistically significant between the two groups (p-value > 0.05).

Another similar study from Taiwan done in 2000 compared EVL and EST for recurrence of varices following endoscopic treatment and its impact on variceal rebleed [10]. From a total of 140 patients, there were 81 episodes of recurrences of varices among 46 (64.8%) patients in EVL group, whereas there were 58 recurrence episodes in 40 (57.1%) patients in EST group. Within two years, the variceal recurrence was more common in ligation than in sclerotherapy and the frequency decreased thereafter. He concluded that early recurrence of varices was more common in EVL than EST group. In our study, only two patients had recurrence of varices, one in each group and the analysis was not statistically significant. We did only one follow-up endoscopy at three months to look for recurrence which would have contributed to the significant difference in the recurrence of varices from the above study in which patients were followed up for two to six years.

An American meta-analysis study done in 2005 compared combination EVL and EST with EVL alone for the secondary prophylaxis of esophageal variceal haemorrhage [11]. They compared eight studies and concluded that addition of EST to EVL does not decrease the recurrent variceal bleed rate or the time taken for eradication of varices. The study revealed that it only increased the risk of stricture formation in the study population. In the present study, there was no difference between the recurrent bleed rates between the two groups.

Another meta-analysis done by Dai et al. from China revealed that EVL is superior in comparison to EST regarding the lower rates of rebleeding, complications, and the higher rate of eradication of varices. They analysed 14 studies consisting of 1236 patients. The rate of rebleeding and rate of variceal eradication in actively bleeding varices patients undergoing EVL was significantly lower (RR = 0.68, 95% CI: 0.57-0.81) and higher (RR = 1.06, 95% CI: 1.01–1.12) respectively in comparison to EST group. The complications rate was significantly lower in EVL group as compared to the EST group (RR = 0.28, 95% CI: 0.13-0.58) [12].

## Conclusions

In the present study, it was found that the EVL alone was effective than ST in terms of number of sessions needed for eradication of varices and total duration required to completely obliterate the varices. The complications were found to be less in EVL group.

The sample size and the follow-up period (three months) of the present study were less. Because of the short-term follow-up period, development of complications like portal hypertensive gastropathy, fundal varices and esophageal stricture could not be evaluated adequately.
